# Fetal cholinergic anti-inflammatory pathway and necrotizing enterocolitis: the brain-gut connection begins *in utero*

**DOI:** 10.3389/fnint.2013.00057

**Published:** 2013-08-08

**Authors:** L. Garzoni, C. Faure, M.G. Frasch

**Affiliations:** ^1^CHU Sainte Justine Research Center, MontrealQC, Canada; ^2^Division of Gastroenterology, Hepatology and Nutrition, CHU Sainte-Justine, MontrealQC, Canada; ^3^Department of Obstetrics and Gynaecology, University of MontrealMontreal, QC, Canada

**Keywords:** necrotizing enterocolitis, chorioamnionitis, preterm birth, inflammation, neuroimmunology, vagus nerve, intestines, prevention

## Abstract

Necrotizing enterocolitis (NEC) is an acute neonatal inflammatory disease that affects the intestine and may result in necrosis, systemic sepsis and multisystem organ failure. NEC affects 5–10% of all infants with birth weight ≤ 1500 g or gestational age less than 30 weeks. Chorioamnionitis (CA) is the main manifestation of pathological inflammation in the fetus and is strong associated with NEC. CA affects 20% of full-term pregnancies and upto 60% of preterm pregnancies and, notably, is often an occult finding. Intrauterine exposure to inflammatory stimuli may switch innate immunity cells such as macrophages to a reactive phenotype (“priming”). Confronted with renewed inflammatory stimuli during labour or postnatally, such sensitized cells can sustain a chronic or exaggerated production of proinflammatory cytokines associated with NEC (two-hit hypothesis). Via the cholinergic anti-inflammatory pathway, a neurally mediated innate anti-inflammatory mechanism, higher levels of vagal activity are associated with lower systemic levels of proinflammatory cytokines. This effect is mediated by the *α*7 subunit nicotinic acetylcholine receptor (*α*7nAChR) on macrophages. The gut is the most extensive organ innervated by the vagus nerve; it is also the primary site of innate immunity in the newborn. Here we review the mechanisms of possible neuroimmunological brain-gut interactions involved in the induction and control of antenatal intestinal inflammatory response and priming. We propose a neuroimmunological framework to (1) study the long-term effects of perinatal intestinal response to infection and (2) to uncover new targets for preventive and therapeutic intervention.

## Introduction

In this review we summarize the emerging understanding of the mechanisms of necrotizing enterocolitis (NEC; Table 1) of the neonate and the clinical significance of the cholinergic anti-inflammatory pathway (CAP) as a neuroimmunological mechanism to prevent NEC. We propose how this emerging understanding of neuroimmunological basis for the NEC etiology may lead to new avenues of clinical research that could result in low cost widely available treatment strategies of NEC across the world. Overall, this review provides a comprehensive, up-to-date review of the literature about the role of CAP in the pathogenesis of NEC as a neuroimmunological modulation system and the emerging therapeutic strategies.

**Table 1:** **Abbreviations**.

CAP	Cholinergic anti-inflammatory pathway
CNS	Central nervous system
ENS	Enteric nervous system
LPS	Lipopolysaccharide
*α*7nAChR	*α*7 subunit nicotinic acetylcholine receptor
NEC	Necrotizing enterocolitis
TNF-*α*	Tumor necrosis factor alpha

## The burden of NEC

NEC is an acute neonatal inflammatory disease that affects the intestine and may result in necrosis, systemic sepsis and multisystem organ failure. The incidence varies from 0.3 to 2.4 infants per 1000 live births, with nearly 90% of cases occurring in infants born at less than 36 weeks’ gestation. NEC affects 5–10% of all infants with birth weight ≤ 1500 g or gestational age less than 30 weeks, and 2–5% of all preterm neonates (Lin and Stoll, 2006). NEC accounts for up to 8% of all admissions to the neonatal intensive care unit. Moreover, with the increased survival of very preterm infants with birth weights ≤ 800 g, the incidence of NEC has increased, despite surfactant replacement therapy (Lin et al., 2008). NEC is the leading cause of death and long-term disability from gastrointestinal disease in preterm infants. Overall mortality ranges from 10–50%, approaching 100% in infants with the most severe form of the disease, which is characterized by full-thickness destruction of the intestine leading to intestinal perforation, peritonitis, bacterial invasion, sepsis and multiorgan failure.

A NEC diagnosis in the very low birth weight infant poses a significant burden to the individual family and the neonatal community, as well as a serious financial burden to society as a whole. NEC is not only one of the most serious clinical problems in neonates, but also one of the most challenging to treat. Because the etiology and underlying mechanisms are unknown, treatment is symptomatic. Minor cases and early stages are managed with antibiotics and cessation of oral feeding; advanced cases, marked by intestinal necrosis, may require intestinal resection.

Despite several decades of research on the pathogenesis of NEC (Hunter et al., 2008), the overall mortality rate remains high and our overall understanding of its causes remains low. Clearly, a more complete understanding of the causes of NEC is required to design more effective and widely affordable preventive strategies aimed at reducing the incidence of NEC as well as therapies (Bisquera et al., 2002; Ganapathy et al., 2012).

## Chorioamnionitis: pathological fetal inflammation is a risk factor

The events leading to NEC are complex and multifactorial, including preterm birth, complicated early neonatal trajectory, adverse intrauterine environment and poor perinatal transition. The most important ones are preterm birth and a history of enteral feeding (Lin and Stoll, 2006). Chorioamnionitis is the main manifestation of pathological inflammation in the fetus and affects 20% of term pregnancies and up to 60% of preterm pregnancies and is commonly an occult finding (Lahra and Jeffery, 2004; Gotsch et al., 2007). Chorioamnionitis is histologically defined by the presence of polymorphonuclear infiltrates in the placenta and its membranes. Even silent, asymptomatic inflammation may inhibit placental angiogenesis and thus modulate the course of the pregnancy (Garnier et al., 2008). Thus, a significant number of fetuses are exposed to various degrees of inflammation, which impacts on their intestinal development. Chorioamnionitis associated with maternal infection has been strongly implicated in fetal intracerebral hemorrhage (Andrews et al., 2006, 2008; Aziz et al., 2009). However, an increased incidence of NEC has also been reported in neonates of mothers presenting with chorioamnionitis, in several independent studies (Andrews et al., 2006; Aziz et al., 2009; Been et al., 2009) as well as a recent meta-analysis (Been et al., 2013), where clinical chorioamnionitis (OR 1.24; 95%, CI 1.02–1.52) and histological chorioamnionitis with fetal involvement (CI 3.29; 95%, OR 1.87–5.78) were significantly associated with NEC. However, the association of histological chorioamnionitis with NEC was not statistically significant. The role of prenatal infection in the development of NEC is most significant in very preterm infants (24–26 weeks’ gestational age) (Aziz et al., 2009), but is also apparent in preterm births < 32 weeks (Andrews et al., 2006; Been et al., 2009) and in full-term births (Martinez-Tallo et al., 1997).

## Tight junctions: intestinal permeability and integrity

**Figure 1:**
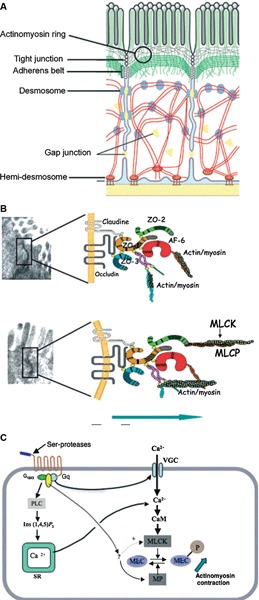
**Structures and factors controlling paracellular permeability**. Tight junctions are luminal structures that filter the passage of ions and macromolecules **(A)**. The intercellular structural proteins, claudins and occludins, are anchored to the cytoskeleton actinomyosin apical ring through intermediary proteins (ZO-1, -2, -3, AF6). The phosphorylation of myosin light chain causing contraction is associated with opening of tight junctions and junction protein deformations that favour macromolecule passage and bacterial translocation **(B)**. The phosphorylation/dephosphorylation of myosin light chain is controlled by myosin light chain kinase (MLCK) and myosin phosphatase (MP). PAR-2 activation increases MLCK activity through external and internal calcium (Ca++) mobilization and calmodulin binding **(C)**. (With permission from John Wiley and Sons) (Bueno and Fioramonti, 2008).

After birth, the intestinal lumen is subject to external environmental influences, including bacterial colonization of the gastrointestinal tract (Gronlund et al., 1999). Cells that cover the intestinal surface must form a barrier to protect the “*milieu intérieur*” from the external world and prevent unrestricted exchange of materials. The intestinal epithelial barrier needs to allow the passage of water and nutrients but prevent microbial contamination and the invasion of interstitial tissues by foreign antigens (Figure 1) (Turner, 2009). Tight junctions, essential to the paracellular pathway, are the primary determinants of barrier function. Tight junctions are situated at the apical pole of epithelial cells, and comprise over 50 associated proteins. The first group includes claudins (a family of at least 24 members) and occludin. These proteins span the plasma membrane and are attached to a second group of proteins including zonula occludens (ZO) -1, -2 and -3, which link them to actin and myosin in the cytoskeleton (Turner, 2009).

The properties of tight junctions differ from one tissue to another, as evidenced by the 1000-fold variation in electrical resistance between different epithelia. Barrier properties are not fixed: they can be modulated and regulated on a short- or long-term basis. Short-term regulation is achieved by deformation of the cytoskeleton: myosin light chain phosphorylation permits interaction with actin and contraction of the actin filaments in the perijunctional ring. Contraction of the actin filaments favours the passage of macromolecules by opening the pores. Myosin light chain kinase-1 (MLCK-1) controls the phosphorylation/dephosphorylation of the myosin light chains in the villus enterocytes and surface colonocytes (Clayburgh et al., 2004). Further downstream, during alterations of the tight junction permeability, the transmembrane protein occludin is subject to endocytosis (Schwarz et al., 2007; Turner, 2009). Long-term regulation of intestinal permeability depends on the synthesis and trafficking of claudin-2, a molecule that is overexpressed in intestinal cells of animal models of colitis and in human ulcerative colitis specimens (Heller et al., 2005).

It has been suggested that abnormalities in intestinal permeability may be key to the facilitation of intestinal inflammation leading to NEC (Petrosyan et al., 2009). Intestinal barrier integrity and its appropriate regulation are essential to the prevention of antigen diffusion and bacterial contamination into the lamina propria and interstitial tissue. McElroy et al. (2012) however, have recently suggested an alternate “bottom up” hypothesis whereby NEC may be caused by injury to the Paneth cells located in the crypts of Lieberkühn (McElroy et al., 2012).

## Intestinal permeability is influenced by pathogens and inflammation

Intestinal barrier dysfunction in newborns may be triggered by exogenous agents. Such enteric pathogens trigger local inflammatory responses through specific receptors (e.g., toll-like receptor 4 (TLR4) for lipopolysaccharide (LPS) recognition) or by proinflammatory cytokines (e.g., tumour necrosis factor alpha (TNF-*α*) (Clayburgh et al., 2005), interferon-*γ* (Wang et al., 2005), interleukin (IL) 1-*β* (Al-Sadi et al., 2008) and high-mobility group box 1 (HMGB1) protein (Sappington et al., 2002; Liu et al., 2006; Raman et al., 2006). TNF-*α*-induced barrier loss is associated with increased transcription and translation of MLCK (Clayburgh et al., 2005). In vivo, intestinal epithelial MLCK is induced by TNF-*α*. In the rat model of sepsis, an intraperitoneal injection of LPS demonstrably resulted in a rapid rise of TNF-*α* in the colonic mucosa followed by an increase in myosin light chain phosphorylation and colonic permeability (Moriez et al., 2005). Interestingly, studies in the fetal rat model of NEC have also shown that prenatal bacterial LPS exposure alters the development and permeability of intestinal epithelium (Giannone et al., 2006) and increases ileal injury (Giannone et al., 2009). Similarly, in fetal sheep, preterm intra-amniotic LPS exposure induces abnormal expression of ZO-1 in the ileum (Wolfs et al., 2009).

## ENS controls epithelial barrier function

The enteric nervous system (ENS) is defined as the arrangement of neurons and supporting cells throughout the gastrointestinal tract, from the esophagus to the anus (Goyal and Hirano, 1996). The ENS is organized in ganglia that contain neurons, glial cells and interstitial cells. Each enteric ganglion contains many different neuron types (Furness, 2000). The ENS consists of some one hundred million neurons, or about one-tenth of the number of neurons of the spinal cord. Glial cells in the ENS have similar properties to those in the central nervous system (CNS) (Gershon et al., 1993). The numbers and types of neurotransmitters expressed by enteric neurons are comparable to those found in the CNS. The ENS is capable of autonomy with elicitation of reflexes (complete reflex circuitry in the intestinal wall comprises intrinsic sensory neurons, interneurons and intrinsic motor neurons) after total extrinsic denervation of the gut (Furness et al., 1995). However, the ENS is under physiological influence of the sympathetic and vagus nerves. The ENS controls intestinal motility, modulates visceral sensation and plays a role in the regulation of the intestinal blood supply and the secretion of digestive hormones (Costa and Brookes, 1994; Kunze and Furness, 1999; Boeckxstaens, 2002). It also plays a major role in water and electrolyte transport. As a consequence, intestinal permeability is under neural control (Keita and Soderholm, 2010). The ENS should thus be considered, along with the microflora, immune system and fibroblasts, as a major player in the maintenance of intestinal homeostasis and integrity. The ENS has the ability to fine-tune intestinal barrier function via the release of mediators such as acetylcholine that enhance— via muscarinic receptors—(Cameron and Perdue, 2007) and vasoactive intestinal peptide (VIP) that constrict (Neunlist et al., 2003) intestinal permeability over short-term or long-term periods (Neunlist et al., 2012). Similarly, the ENS can modulate the proliferation and differentiation of the intestinal epithelial barrier via the secretion of distinct neuromediators such as VIP (Neunlist et al., 2012). VIP exerts antiproliferative effects, while mediators such as acetylcholine, glucagon-like peptide 2 (GLP-2) or substance P stimulate intestinal epithelial cell proliferation (Neunlist et al., 2012).

## NEC: immature immune response

Local intestinal immune response is normally tightly controlled (Su et al., 2009).

The premature gastrointestinal tract has increased permeability, low levels of protective mucus and secretory immunoglobuline A, higher risk of bacterial overgrowth caused by dysmotility due to ENS immaturity and decreased regenerative capabilities (Neu, 2007). Uncontrolled intestinal inflammation may result from immaturity of the innate immune system of the developing gut (Lin and Stoll, 2006; Lin et al., 2008). Immature regulation could lead to an exaggerated inflammatory response, leading to greater injury and increased intestinal barrier damage. Alternatively, immature regulation could result in minimal immune response due to insufficient inflammatory signalling, thus contributing to bacterial overgrowth and invasion of interstitial tissue. The uncontrolled intestinal inflammation observed in NEC may also depend on dysregulation of intestinal permeability in relation to localized immune response (Turner, 2009). In most individuals and specifically in healthy full-term newborns, a localized break in the intestinal barrier induces a localized immune response that is finely tuned and controlled to avoid over-inflammation and a subsequent increase of intestinal permeability. This normal immunoregulatory response is the result of a delicate balance between pro-inflammatory (TNF-*α*, IL-1*β*) and anti-inflammatory (IL-10) processes. If even small anomalies occurred in any of the components of the system (tight junction dysregulation, immune regulatory response), the inflammatory response would be amplified and would result in intestinal injury. Such anomalies may occur secondary to immaturity in preterm babies.

Furthermore immunomodulatory nutrients such as glutamine, arginine, nucleotides, omega-3 polyunsaturated fatty acids and lactoferrin are provided with enteral nutrition and prevent diseases such as NEC. Difficulties with enteral feeding in the first weeks of life predispose premature infants to sepsis and NEC (Neu et al., 2013).

## Development and monitoring of the autonomic nervous system activity

The autonomic nervous system (ANS) plays a predominant role in complex coordinated control of multiple vitally important physiological subsystems in the organism and is part of the neuroimmunological response to pathogens via CAP (Fairchild et al., 2011). Since ANS development and activity are reflected in the heart rate patterns, an appropriate analysis of the fetal heart rate variability (fHRV) may provide information regarding the individual fetal development (Hoyer et al., 2013; Van Leeuwen et al., 1999). fHRV is a non-invasively obtainable marker of changes in vagal (parasympathetic) and sympathetic activity (Frank et al., 2006). Increase in fHRV is associated with fetal growth in general and with the increase in neural integration in particular (Van Leeuwen et al., 2013). Understanding of the dynamics of fHRV in human and ovine fetuses during physiologic (e.g., sleep states) and pathophysiologic (e.g., asphyxia) conditions has evolved over the past two decades (Karin et al., 1993; Frank et al., 2006; Shapiro et al., 2013). Fetal heart rate (FHR) and fHRV are regulated by a complex interplay of the parasympathetic and sympathetic nervous systems accounting for the baseline FHR as well as short-term and long-term variability and nonlinear properties (Frasch et al., 2009; Gieraltowski et al., 2013). Nonlinear properties of fHRV in late gestation fetuses are present and a higher vagal tone is associated with more efficient regulation of homeostasis (Groome et al., 1999).

## CAP controls immune homeostasis

**Figure 2:**
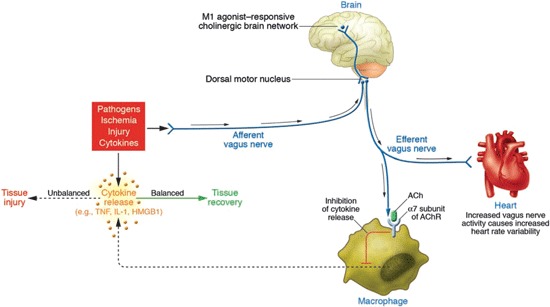
**Wiring of the cholinergic anti-inflammatory pathway (CAP), which balances cytokine production**. Pathogens as well as ischemia and other forms of injury activate cytokine production, which normally restores health. If the cytokine response is excessive, however, then these same mediators can cause disease. Efferent signals from the vagus nerve inhibit cytokine production via pathways dependent on the *α7 subunit* of the acetylcholine receptor (*AChR*) on macrophages and other cells. Efferent vagus nerve activity also increases instantaneous heart rate variability (HRV). A cholinergic brain network that is responsive to M1 agonists can increase the activity of CAP and also increase instantaneous HRV. Afferent signals carried in the vagus nerve can activate an efferent response that inhibits cytokine release, termed the *inflammatory reflex* (With permission from American Society for Clinical Investigation) (Tracey, 2007).

**Figure 3:**
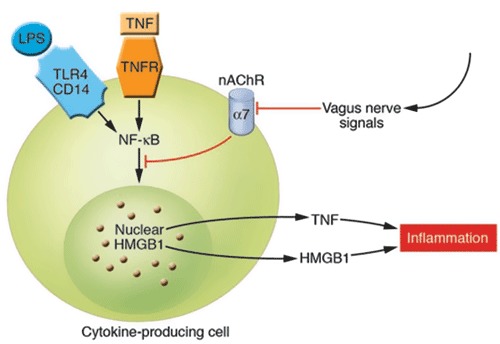
**Cholinergic signals derived from vagus nerve stimulation inhibit the release of TNF-*α*, IL-1, HMGB1, and other cytokines by transducing a cellular signal that inhibits the nuclear activity of NF-κB**. TNFR stands for TNF receptor (Wang et al., 2004; Tracey, 2007). The cytokine producing cell can be a macrophage, among others. The nAChR family are ligand-gated ion channels that mediate diverse physiological functions and were originally identified in the nervous system. They consist of different subtypes formed by the specific assembly of five polypeptide subunits including *α*1-10, *β*1-4, *γ*, *δ*, and *ε*. The subunits fall into two groups: neuronal nicotinic receptors (consisting of *α*2–10 and *β*2–4) and muscle nicotinic receptors (consisting of *α*1, *β*1, *γ*, *δ*, and *ε*). Functional neuronal nAChR subtypes are either homomeric (consisting of 5 identical *α*-subunits, as in *α*7- or *α*9 nAChR) or heteromeric (consisting of combinations of the *α*- and *β*-subunits, such as *α*3*β*2nAChR) (Yeboah et al., 2008). Remarkably, it is specifically the *α*7nAChR that is required for CAP’s effects on peripheral innate immune cells (Figures 2,**3**) (With permission from American Society for Clinical Investigation) (Tracey, 2007).

CAP has been implicated in the regulation of the inflammatory reflex in adult organisms including humans (Tracey, 2002, 2007, 2009; Cailotto et al., 2012; Olofsson et al., 2012). CAP is a neural mechanism that influences the magnitude of the innate immune response and maintains homeostasis. As part of CAP, increased vagal activity inhibits the release of proinflammatory cytokines (Figures 2,3). Vagal nerve stimulation decreases LPS-induced systemic TNF-*α* release in adult rats (Borovikova et al., 2000). Suppression of proinflammatory cytokine expression via agonistic action on the *α*7 subunit nicotinic acetylcholine receptor (*α*7nAChR) was confirmed in innate immune cells such as macrophages (reviewed in Tracey, 2007) (Figures 2,3). Systemically via CAP, this is mediated via the spleen: Adrenergic nerve fibres in the spleen activate acetylcholine-producing T-lymphocytes, thereby inhibiting systemic cytokine production (Huston et al., 2007; Rosas-Ballina et al., 2011; Olofsson et al., 2012).

Vagal nerve activity in basal conditions provides inhibitory input that dampens innate immune response (Haensel et al., 2008; Thayer and Fischer, 2009). The CAP influences the magnitude of the innate immune response and maintains homeostasis (Tracey, 2009). Depressed vagal nerve activity is associated with an exaggerated proinflammatory response and increased morbidity and mortality in various contexts such as acute or stable coronary heart disease, metabolic syndrome or impaired glucose tolerance and kidney failure (Haensel et al., 2008; Thayer and Fischer, 2009). The inhibitory role of CAP on innate immune function can be thought of as analogous to the inhibitory role of the vagus nerve on the resting heart rate (Tracey, 2009).

**Figure 4:**
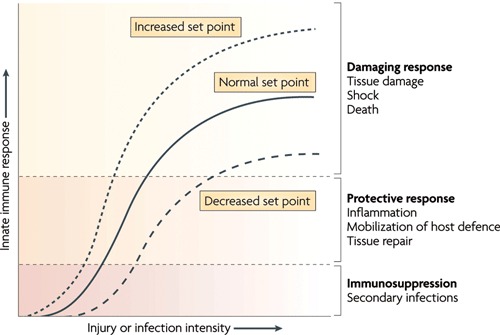
**The set point function of the immune response is defined by the magnitude of innate immune responses relative to the infection or injury stimulus**. Increasing the set point or shifting the curve to the left increases the chance that tissue damage will occur from the response to infection or injury. Decreasing the set point or shifting the curve to the right reduces the probability that tissue damage will occur. CAP is the neural circuit that provides acute compensatory input to adjust the magnitude of the immune response relative to the set point (With permission from Nature Publishing Group) (Tracey, 2009).

Under resting conditions, the inflammatory reflex helps establish the set point for the magnitude of the innate immune response to molecules arising from infection, injury or ischemia. Vagus nerve output maintains homeostasis by limiting proinflammatory response to the healthy, protective and non-toxic range ( Figure 4). However, when vagal activity is absent or diminished, the set point increases; exposure to pathogens then results in an exaggerated proinflammatory response and eventual tissue damage as demonstrated in different experiments with murine models (Ghia et al., 2006, 2007a,b, 2008).

Numerous factors can experimentally or clinically impair the CAP, each resulting in an exaggerated innate immune response. For instance, in animal models with vagotomy or deficient in *α*7nAChR, the magnitude of the proinflammatory cytokine response and the extent of tissue damage increase during infection, haemorrhagic shock and stroke (Guarini et al., 2003; van Westerloo et al., 2005; Ghia et al., 2008; Ottani et al., 2009; Tracey, 2009). The observation that vagal nerve activity influences circulating TNF-*α* amounts and the shock response to endotoxemia has widespread implications. It is a previously unrecognized, direct and rapid endogenous mechanism that can suppress the lethal effects of biological toxins. The CAP has much shorter response times than humoral anti-inflammatory pathways. Electrical stimulation of the vagus nerve or administration of *α*7nAChR agonists reduces the magnitude of proinflammatory cytokine production by 50–75% but does not eliminate cytokine activity (Borovikova et al., 2000; Tracey, 2009). Activation of CAP has not been observed to cause immunosuppression because maximal suppression only reduces proinflammatory cytokine levels from the toxic to the healthy range (Figure 4). This concept has been studied as a potential treatment for a range of inflammatory diseases, including infection (reviewed in Tracey, 2009). The role of CAP in the perinatal inflammatory response and the priming of subsequent innate immune response require elucidation.

Adult clinical data indicate that loss of inhibition from the CAP unleashes innate immunity, produces higher levels of proinflammatory mediators, and exacerbates damage to the organism (Lindgren et al., 1993; Lanza et al., 2006; Thayer and Sternberg, 2006; Marsland et al., 2007; Sloan et al., 2007; Tateishi et al., 2007; Haensel et al., 2008; Holman and Ng, 2008; von Kanel et al., 2008) and exacerbation of tissue damage. Studies are needed to explore the impact of CAP activity on chorioamnionitis (Shapiro et al., 2013). Such studies could lead to the development of novel prognostic markers to better identify fetuses at risk of NEC. We hypothesize that increased CAP activity would inhibit the activation of intestinal innate immune cells such as macrophages and thus suppress the inflammatory response to bacterial infection. Effectively, pathological inflammation and intestinal permeability as a *locus minoris resistentiae* of incipient NEC would be decreased or normalized.

### Vagal nerve stimulation and the gut inflammation

As vagal nerve stimulation stimulates CAP activity without causing immunosuppression (Borovikova et al., 2000), it has been shown to improve intestinal inflammation in murine models of experimental colitis (Ghia et al., 2006, 2007a,b, 2008) and postoperative ileus (The et al., 2007; van Bree et al., 2012). The role of intestinal macrophages seems pivotal (The et al., 2007; Costantini et al., 2010a; Rosas-Ballina et al., 2011; van Bree et al., 2012). In a murine model of dextran sodium sulphate-induced colitis, Ghia et al. demonstrated an increased inflammatory response in the colonic mucosa of the vagotomy group as compared to control animals. They showed the protective effect of vagal activity in acute colitis (Ghia et al., 2006) and in acute relapses within a background of chronic inflammation (Ghia et al., 2007b).

In a murine model of postoperative ileus, the anti-inflammatory effect of intracerebroventricular injection of semapimod was abolished in the presence of vagotomy (The et al., 2011). Vagal nerve stimulation also modulates intestinal permeability and integrity. In a murine model of intestinal injury caused by severe burns, vagal nerve stimulation performed before injury improved intestinal barrier integrity through an efferent signalling pathway and was associated with improved tight junction protein expression (Costantini et al., 2010b). In the same model, stimulating the vagus nerve at the time of injury promoted enteric glial cell activation. Either method could prevent intestinal barrier injury (Costantini et al., 2010a). Activation of enteric glial cells has been reported in a rat model of burn-induced stress and gut injury (Costantini et al., 2010a).

The optimal choice of vagal nerve stimulation parameters in order to activate the CAP for therapeutic purposes remains challenging. Although the literature provides a spectrum of possibilities, further studies are needed. Huston et al., 2007 demonstrated improved survival in murine polymicrobial sepsis with transcutaneous vagal nerve stimulation (Huston et al., 2007), suggesting that this mode might be an effective therapy for sepsis generally and NEC specifically. Pharmacologically, cholinergic neuronal circuitry can be stimulated peripherally using *α*7nAChR agonists to act on macrophages; or centrally using intracerebroventricular injections of semapimod (The et al., 2011), muscarinic receptor agonist McN-A-343 (Pavlov et al., 2006), or an acetylcholinesterase inhibitor (galantamine) (Pavlov et al., 2009). Importantly, enteral feeding of lipid-rich nutrition may also be used to activate the CAP pathway (Luyer et al., 2005).

### Significance and novelty

Intrauterine infection leads to macrophage activation in LPS-induced chorioamnionitis in fetal sheep and in a rat model of postnatal sepsis. Intrauterine infection may prime innate immune cells for subsequent exacerbated response and increase intestinal permeability. However, the effects of CAP on this process have not been elucidated. The existence of CAP in adult mammals, including humans, is by now well established. CAP may play an important role in fetal and perinatal development by serving as a neuroimmunological network for “internal surveillance” linking the CNS and the vagus nerve to modulate systemic and intestinal inflammation. The presence of such a brain-gut network and its significance for perinatal health have not yet been fully studied. Further research on the physiology and pathophysiology of fetal and perinatal neuroimmunological interactions may open new avenues for diagnosis of fetuses at risk of intestinal injury, such that appropriate preventive or therapeutic interventions may be taken. Specifically, fetal CAP activation is likely to suppress the adverse effects of macrophage activation thus decreasing antenatal and perinatal intestinal injury. New pharmacologic targets or validated non-invasive methods of vagus nerve stimulation for manipulating the inflammatory response in compromised fetuses may emerge to improve perinatal and postnatal outcome.
